# Targeting the Pim kinases in multiple myeloma

**DOI:** 10.1038/bcj.2015.46

**Published:** 2015-07-17

**Authors:** N A Keane, M Reidy, A Natoni, M S Raab, M O'Dwyer

**Affiliations:** 1 Apoptosis Research Centre, National University of Ireland Galway and Department of Haematology, Galway University Hospital, Galway, Ireland; 2 Apoptosis Research Centre, National University of Ireland Galway, Galway, Ireland; 3 Max-Eder Unit “Experimental Therapies for Hematologic Malignancies”, Department of Medicine V, Heidelberg University Medical Center, Im Neuenheimer Feld 410 and German Cancer Research Centre (DKFZ), Heidelberg, Germany

## Abstract

Multiple myeloma (MM) is a plasma cell malignancy that remains incurable. Novel treatment strategies to improve survival are urgently required. The Pims are a small family of serine/threonine kinases with increased expression across the hematological malignancies. Pim-2 shows highest expression in MM and constitutes a promising therapeutic target. It is upregulated by the bone marrow microenvironment to mediate proliferation and promote MM survival. Pim-2 also has a key role in the bone destruction typically seen in MM. Additional putative roles of the Pim kinases in MM include trafficking of malignant cells, promoting oncogenic signaling in the hypoxic bone marrow microenvironment and mediating resistance to therapy. A number of Pim inhibitors are now under development with lead compounds entering the clinic. The ATP-competitive Pim inhibitor LGH447 has recently been reported to have single agent activity in MM. It is anticipated that Pim inhibition will be of clinical benefit in combination with standard treatments and/or with novel drugs targeting other survival pathways in MM.

## Introduction

The Pim kinases have emerged as an attractive novel therapeutic strategy in the hematologic malignancies in general, and in multiple myeloma (MM) in particular. Pims are constitutively expressed uniquely in the hematologic malignancies and Pim-2 expression is higher in MM than in any other cancer or in physiology.^1^ Established roles for the Pim kinases in MM are diverse and include MM proliferation,^1^ survival,^2^ cell cycle dysregulation,^3, 4^ an oncogenic collaboration with the most frequently dysregulated gene in MM (Myc)^5, 6^ and mediating bone destruction.^7^ Putative roles include mediating drug resistance, migration and homing of MM cells. The rationale for targeting the Pims in MM, lead Pim inhibitors in development and the potential application of Pim inhibition in treatment of MM are discussed herein.

## Background—Pim kinases

The Pim family of serine/threonine kinases are named for their mode of discovery as proviral common integration sites in moloney murine leukemia virus (mMuLV)-induced lymphomas.^8^ Insertional mutagenesis screening utilizes transforming retroviruses to identify oncogenes overexpressed by the activity of the retroviral enhancer sequence.^9^ Cloning of retroviral integration sites in mMuLV-induced lymphomas led to the discovery of Pim-1 in the 1980s^8^ followed by Pim-2^10^ and later Pim-3 in the 1990s in the screening of Pim-1/Pim-2 knockout models.^11^

**Figure 1:**
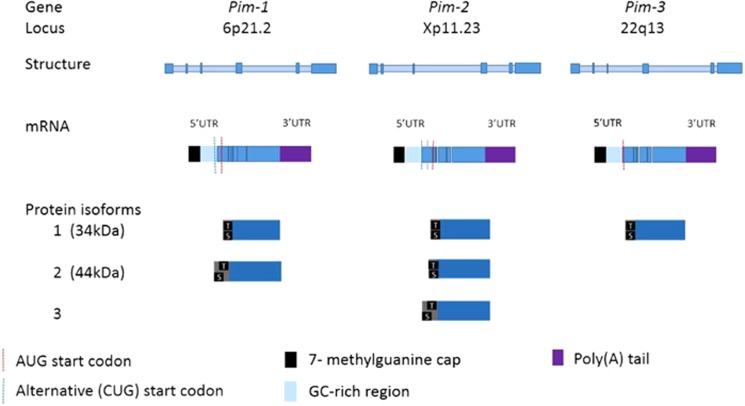
Genetic structure of the Pims. The Pim kinases share significant homology (>60%).^18^ Each Pim gene contains 6 exons (depicted in darker blue). Pim mRNA contains a 5' untranslated region (UTR) which is comprised of a 7 methyl-guanine cap and GC-rich region which renders the Pims ‘weak transcripts' requiring cap-dependent translation.^26^ The 3' UTR contains destabilizing AUUUA motifs which result in a short Pim mRNA half life.^25^ Pim AUG start codons are located at nucleotides 431–433 and result in translation of one and two longer isoforms of Pim-1 and Pim-2, respectively.^19^ The longer 44kDa isoform of Pim-1 is derived from use of an upstream CUG start codon at nucleotides 158–160 and localizes to the plasma membrane, with a role in chemotherapeutic resistance.^19^ Pim proteins are autophosphorylated at an upstream serine 8 residue. A threonine residue and two downstream serine residues are also present. There is no regulatory domain and the overlapping catalytic and ATP-binding domains constitute the majority of the Pim proteins.

The Pim family is highly conserved with greater than 60% homology between each member^12^ and the genetic structure is outlined in Figure 1. Pims lack a regulatory domain and thus are constitutively active.^13, 14^ Pims have a unique structure divergent from that of other kinases with two proline residues located in the hinge region.^13^ Only one hydrogen bond is formed with ATP, with implications for drug development as the majority of ATP-competitive inhibitors form two. The *K*_m_ (concentration of substrate that leads to half maximal velocity) of Pim-2 for ATP is up to 100-fold lower than that of Pim-1 and Pim-3^15^ making a pan-inhibitor more challenging to develop than specific inhibitors.^15, 16^

Regulation of Pim kinases is predominantly transcriptional and translational. Growth factor signaling via the janus kinase/signal transducer and activator of transcription (JAK-STAT)^17^ and nuclear factor Kappa-light-chain-enhancer of activated B cells (NFκB)^18^ pathways results in transcription of Pim mRNA. STAT3 and STAT5 levels correlate with Pim expression.^14^

Pim mRNA has a short half-life because of the presence of the destabilizing AUUU(A) sequence at the 3' region, though in hematologic cancers longer half-lives are observed.^19^ The Pim 5' region is GC sequence-rich and hence is a ‘weak transcript' requiring cap-dependent translation (see section 'Pim kinases and cancer—Cap-dependent translation').^20^ Pims are capable of autophosphorylation at serine8,^21^ but the stability of the transcribed PIM proteins is the key regulator of Pim activity. Pims are dephosphorylated, ubiquitylated and directed for proteasomal degradation by the B56β protein phosphatase 2A (PP2A).^22^ Members of the heatshock protein family have opposing roles in the regulation of Pim activity with HSP90 stabilizing Pim levels and HSP70 marking Pims for ubiquitylation and proteasomal degradation.^23^

## Pim knockout studies

Initial studies involving knockout of all three Pim kinases (*Pim1−/− Pim2−/− Pim3−/−*) resulted in mild phenotypic changes in murine studies. Mice were viable, fertile but exhibited reduced body size.^24^

Deficient Pim-1 signaling has since been linked to impaired cardiac functioning.^25, 26, 27^ Reduced Pim-1 expression in diabetic mice results in onset of heart failure which is reversed with restoration of Pim-1.^25^ Pim-1 may exert a cardioprotective function by maintaining a pool of functional cardiomyocyte mitochondria thereby preventing cardiomyocyte aging.^26^ Indeed increased myocardial repair is noted in heart failure patients treated with human cardiac progenitor cells engineered to express PIM1.^27^ Pim triple knockout mice in later studies developed heart failure by 6 months.^26^ These observations regarding the importance of Pims in cardiac function proved relevant in the clinic with first generation Pim inhibitor SGI-1776 withdrawn from early stage clinical investigation owing to cardiotoxicity.

## Pim kinases and cancer

Pim kinases are widely expressed in cancer with higher expression in hematologic than solid organ malignancies.^1^ Pim-2 is most highly expressed in MM, while Pim-1 is most highly expressed in acute myeloid leukemia (AML) and chronic myeloid leukemia.^1^ Pim-3, while expressed throughout the hematologic malignancies, shows no distinct pattern of expression favoring a particular malignancy^1^ and bears the least association with hematological malignancies, instead being strongly associated with solid organ malignancies.^28^ Expression of Pim kinases confers a worse prognosis in many malignancies, including AML.^12^

### Key mechanisms by which Pims exert oncogenic effects

#### Oncogenic collaboration with Myc

Myc activity is necessary for Pims to effect oncogenesis,^6^ and in turn, the Pims phosphorylate, stabilize and enhance the transcriptional activity of Myc, an effect greatly diminished by Pim knockdown.^29^ Pim kinases complex with Myc and Myc-associated factor X(MAX), with Myc recruiting Pim to the E-box of target genes.^30^ Pim kinases then phosphorylate serine10 of histone H3 (H3S10) of the Myc binding site nucleasome, increasing transcriptional activation of approximately 20% of Myc-targeted genes and contributing to cellular transformation.^30^ H3S10 phosphorylation facilitates interaction with 14-3-3 proteins which trigger the acetylation of histone H4, and this crosstalk between the histones provides a platform for bromodomain protein binding.^31^ The ensuing recruitment of transcription elongation factor b (TEFb) releases promoter-proximal paused RNA polymerase II and activates transcription.^31^

The originally described mMuLV-induced T-cell lymphomas exhibited highly increased c-MYC expression, with proviral insertions frequently occurring in close proximity to both c-MYC and Pim-1 genes.^32^ Pim-1 transgenic mice with Eμ Immunoglobulin enhancer sequences upstream of the Pim gene and long terminal repeats downstream exhibited greatly increased expression of Pim-1 with a corresponding increase in lymphomagenesis.^33^ Newborn Pim-1 transgenic mice infected with mMuLV with activation of MYC developed T-cell lymphomas with a latency of only 7–8 weeks,^33^ evidencing a very strong oncogenic collaboration between these genes. While Pim-1 and MYC transgenic mice were predisposed to T- and B cell lymphomas, respectively, double transgenic Eμ Pim-1 and Eμ MYC developed an aggressive pre-B leukemia *in utero* with death at or before birth.^34^

Further evidence for this Pim/MYC collaboration and MYC dependence on Pim expression for oncogenesis is provided by observation of longer latency to development of lymphoma in Pim knockout mice.^11^ In support of redundancy of Pims in hematological malignancy, upregulation of Pim-2 in Pim-1-deficient mice and Pim-3 in Pim-1/Pim-2-deficient mice with preserved lymphomagenesis is observed.^11^

#### Cap-dependent translation

Pims are important in the upregulation of proteins involved in cell cycle regulation and cell survival via cap-dependent translation in cancer. Pim-2 phosphorylates tuberous sclerosis complex-2 (TSC2) to derepress mammalian target of rapamycin complex-1 (mTORC1).^1^ mTORC1 then phosphorylates EIF4E-binding protein-1 (4EBP1) and ribosomal protein S6 kinase (S6K).

Phosphorylation of 4EBP1 facilitates separation from EIF4E and allows recruitment to ribosomal subunit 40S of m^7^G-capped mRNA for translation. EIF4E is necessary for Pim-induced cap-dependent translation to occur.^35^ Furthermore, the activation of EIF4E following mTORC signaling is crucial for MYC survival signaling.^36^ The Pims themselves, as well as MYC, cyclin D1, myeloid cell leukemia-1 (MCL-1), important in survival and cell cycle progression, constitute ‘weak' mRNA targets owing to their 5' GC-rich region^37^ and rely on this mechanism of translation.

In B-cell lymphoproliferative malignancies, Pim-2 has a dominant role by regulating mTORC1, as evidenced by reduced phospho-4EBP1 with Pim inhibition.^38^ In chronic lymphocytic leukemia, Pim inhibition at concentrations sufficient to reduce MYC and MCL1 expression affects cell death, whereas antiapoptotic effects were not affected at this level. Similar data are presented relating to MM, indicating a dominant role for blockade of Pim-2-induced cap-dependent translation in clinical use of Pim inhibitors in lymphoid malignancy.^39^

#### Anti-apoptotic activities

The best described anti-apoptotic effect of the Pims is that on Bcl-2-associated agonist of cell death (BAD) phosphorylation. This effect was initially discovered in Pim-2.^14^ Multiple sites on BAD may be phosphorylated to prevent apoptosis,^40^ with S112 phosphorylation, the dominant residue involved in Pim-1 and Pim-2 signaling, and Pim-3 favoring S136 and S155 phosphorylation.^40^ Following phosphorylation, 14-3-3 binding occurs with dissociation of BAD from B-cell Lymphoma-extra large prosurvival protein (Bcl-XL) and relocation from the mitochondrion to the cytosol.^40^ Other anti-apoptotic activities include phosphorylation of murine double minute 2 homolog (MDM2) at serine166 and 186 to prevent proteasomal degradation of p53 in mantle cell lymphoma^41^ and inhibit the apoptotic actions of apoptosis signal-regulating kinase-1 (ASK-1) by its phosphorylation.^42^

#### Cell cycle regulation

The Pim kinases phosphorylate cyclin-dependent kinase inhibitors p21 and p27.^43, 44^ Phosphorylated p21 relocates to the cytoplasm and is stabilized to increase proliferation.^43^ By contrast, Pim phosphorylation of p27 induces 14-3-3 binding, its transport from the nucleus and proteasomal degradation.^44^ Pims also inactivate Forkhead transcription factors to downregulate p27.^44^ Pims target the G1/S transition point by phosphorylating CDC25A, a transcriptional target of Myc and increasing cyclinD1 activity.^4^ Pims regulate the G2/M checkpoint by phosphorylating CDC25C and CDC25C-associated kinase 1.^3^ Pim-1 and -2 have also been found to phosphorylate Chk1 at serine280 in AML,^45^ perhaps as a component of the FLT3/STAT5/PIM/CHK1 axis, though increased Chk1 phosphorylation is also observed in Flt3 wild-type AML primary samples,^45^ and inhibition of Pims in wild-type or mutated Flt3 AML appears to result in similar inhibition of proliferation.

## Targeting the Pim kinases in MM

**Figure 2:**
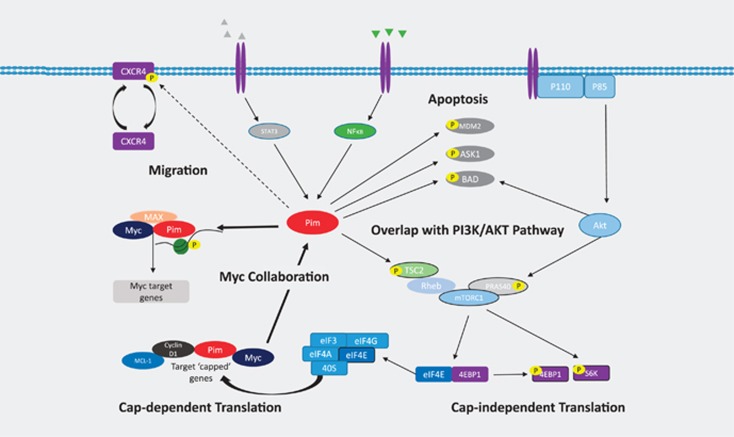
Pim signaling in MM. Pim-2 is upregulated in MM. BMSCs, present in the microenvironment secrete IL-6 (depicted in grey) and increase Pim-2 transcription via STAT3 signaling. OCs release tumor necrosis factor family members TNFα, BAFF and APRIL (depicted in green), which then act via NFκB to increase Pim-2 transcription. Pim-2 has a role in prevention of apoptosis by phosphorylating MDM2 and reducing the degradation of p53, phosphorylation of ASK-1 and phosphorylation of the pro-apoptotic BAD at serine 112. This latter effect on BAD is shared with the PI3K/AKT/mTOR pathway also important in MM. These two pathways also converge on mTOR signaling. Pim-2 phosphorylates TSC2 to release the inhibitory effect of Rheb on mTORC1. Akt phosphorylates PRAS40 to activate mTORC1. Downstream of mTOR activation, both cap-dependent and cap-independent translation is initiated. The ribosomal proteins 4EBP1 and S6 kinase are phosphorylated to initiate cap-independent translation. Following phosphorylation of 4EBP1, eIF4E is released and forms a translation initiation complex with eIF4A, eIF4G and eIF3. The ribosomal 40S subunit can then bind to ‘weak' mRNA transcripts which contain a GC-rich region and capped by 7-methylguanosine. Among the pro-myeloma proteins translated in this manner are MYC, cyclin D1, MCL-1 and the Pim kinases themselves. This forms part of the oncogenic collaboration between the Pims and MYC. The Pims cannot perform oncogenic functions in the absence of MYC expression,^39^ and in turn, the Pims phosphorylate and stabilize MYC.^40^ Pims complex with MYC and MAX and are recruited to the E-box of MYC where Pims phosphorylate serine10 of histone 3 (H3S10) to induce transcription of up to 20% of MYC target genes.^41^ A putative role for the Pim kinases in MM, as has been demonstrated in other hematologic malignancies, is phosphorylation of CXCR4 on serine 339 with resultant internalization and re-expression of CXCR4, facilitating homing and migration.

Pims recently emerged as an exciting new target in MM. High expression of Pim-2 is seen in MM and important in mediating MM cell survival and proliferation, by inhibiting apoptosis and inducing cap-dependent translation, respectively (Figure 2). Furthermore, with Myc dysregulation, the most frequent genetic abnormality encountered in MM,^5^ and strong oncogenic collaboration between the Pims and Myc, Pim inhibitors seem an obvious choice for drug development in MM. In addition, Pims crosstalk with and share significantly overlapping functions with other kinase signaling pathways active in MM.

Pim-2 is more highly expressed in MM than in any other malignancy.^1^ Microarray analyses identify Pim-2 as a key player in B-cell development as well as MM.^46^ High expression is not observed in normal plasma cells and expression increases in MM versus monoclonal gammopathy of unknown significance.^47^ This infers a role for Pim-2 in progression to MM, consistent with observations in other lymphoid malignancies of increasing Pim-2 expression with advancing disease.^38, 48, 49^ At the time of writing, there were no published data to establish a worse prognosis conferred by increased Pim expression in MM, though this has been seen in other lymphoid malignancies.^38^

### Upregulation of anti-apoptotic Pim-2 in MM

Pims are constitutively active following growth factor signaling in MM, with no mutated forms reported to date, though a high rate of somatic hypermutation involving the Pim genes is reported in other B lymphoproliferative malignancies.^48, 49^ The bone marrow microenvironment has a dominant role in upregulation of Pim-2 in MM. Bone marrow stromal cells (BMSCs) and osteoclasts (OCs) elaborate specific growth factors to increase the expression of Pim-2 in MM cells.^2^ BMSCs have a more pronounced effect, with a two to ninefold increase in Pim-2 mRNA following BMSC coculture of MM cells mediated by production of interleukin (IL)-6, which acts via STAT3 to increase transcription of Pim-2.^2^ OCs increase Pim-2 expression via the tumor necrosis factor family members TNFα, BAFF and APRIL, which act by NFκB signaling to increase transcription.^2^ NFκB is necessary for Pim-2 anti-apoptotic effect, and Pim-2 in turn increases phospho-IκB and Cot transcription factor to increase NFκB, in a positive feedback loop.^50^ In support of these mechanisms of Pim-2 upregulation in MM, inhibition of STAT3, NFκB and IL-6 all reduce Pim-2 expression in *in vitro* models.^2^ In contrast to IL-6 and NFκB, IGF-1 has not been found to have a significant role in mediating Pim-2 expression via the microenvironment^2^ as it does with other signaling pathways in MM. Cooperative enhancement is observed with a combination of BMSCs and OCs in the microenvironment increasing Pim-2 expression significantly. Furthermore, siRNA knockdown of Pim-2 significantly abrogates MM cell viability in coculture with BMSCs and OCs,^2^ confirming the anti-apoptotic role of Pim-2 in MM.

### Role of Pim-2 in proliferation and cell cycle progression in MM

Pim-2 is necessary for proliferation in MM.^1^ Pim-2 phosphorylates the negative regulator of mTOR, TSC2, at serine 1798 to relieve its suppressive effect on mTORC1 activity in MM.^1^ Phosphorylation of S6K and 4EBP1, substrates of mTORC1 signaling, increases following Pim-2 signaling, facilitating cap-dependent translation and proliferation by the mechanism already described. There is evidence from preclinical work in MM using Pim inhibitors that inhibiting this process is the key anti-MM effect of these drugs.

Pim inhibition with LGB321 reduces phosphorylated TSC2 and mTORC1 activity.^1^ Pim inhibition with thiazolidine class reduces 4EBP1 phosphorylation and the amount and phosphorylation of MCL1 and c-MYC in MM cell lines.^2^ Reduced anti-apoptotic phospho-BAD is seen following Pim-2 inhibition with this drug, or Pim-2 knockdown, but the effect is less pronouned than that of reduced proliferation.^1^ Pharmacological inhibition with SGI-1776 results in no change in apoptosis or cell cycle regulation,^51^ but protein tranlsation is affected, with reduced phosphorylation of 4EBP1 and P70S6K.^51^ Furthermore, in MM animal models, maximal in vivo effect is dependent on the inhibition of mTORC1 signaling, and hence inhibition of proliferation, being potent and sustained.^1^

### Pim-2 and MM bone disease

An emerging role for Pim-2 in the bony destruction and marrow expansion of MM has been reported.^7^ Pim-2 is seen to negatively regulate osteoblastogenesis in murine cell line models, with the effect reversed on Pim-2 knockdown or pharmacological inhibition.^7^ The chief osteoblastogenic effects mediated by Pim inhibition were increase of osteoblastogenic factor bone morphogenetic protein-2 (BMP-2) and reduced transforming growth factor β signaling. Pim-2 expression is upregulated not only in MM cells but also in BMSCs and osteoblast precursors in the microenvironment in response to known suppressors of osteoblastogenesis IL-3, IL-7, Activin A, TNFα and transforming growth factor β, and this effect was also noted in primary human MM BMSCs following culture with conditioned media.^7^ Pim inhibitors are capable of counteracting these effects to restore osteoblastogenesis. Murine models of human MM treated with Pim inhibitor SMI16a reveal reduced tumor growth, prevention of bony destruction and restoration of bone formation compared to control animals.^7^ The Pim inhibitor LGH447 reduces bone disease in MM xenograft models ^52^ and reduces formation and functionality of OCs.^53^

### Putative role of Pim-1 in homing to the hypoxic MM bone marrow microenvironment

MM is characterized by continuous migration of malignant cells from the bone marrow, and homing to new sites in the marrow.^54^ It has been shown that SDF1α and CXCR4 interactions mediate MM expansion and homing^55^ and that inhibition of CXCR4 inhibits MM homing.^56^ The MM bone marrow microenvironment is a hypoxic niche.^57^ Hypoxia reduces MM adhesion to bone marrow stroma by reducing E-cadherin expression following increasing expression of SNAIL, FOX2C and transforming growth factor 1β.^58^ Hypoxia also results in reduced SDF1α production by stromal cells locally,^58^ potentially encouraging migration and homing to new sites in the marrow. CXCR4 levels are higher in circulating MM cells than in those resident in the bone marrow.^54^ Further, hypoxia induces upregulation of CXCR4 in a HIF1α-dependent manner in MM.^58^ The hypoxic bone microenvironment conditions also promote Pim activity with inhibition of the ubiquitin-mediated proteasomal degradation of Pim.^59^

It has been demonstrated in AML that Pim-1 is important for CXCR4 expression and homing of stem cells. A clear association between blast expression of Pim-1 and surface expression of CXCR4 has been established^60^ and confers a worse prognosis.^61^ Pim-1 phosphorylates Serine339 on CXCR4 facilitating receptor internalization and re-expression on the cell surface.^60^ Conversely, Pim inhibition results in reduced CXCR4 expression.^60^ In murine models, the knockout of Pim-1 results in the inability to reconstitute bone marrow following irradiation, and Pim-1 knockout cells express greatly reduced amounts of CXCR4.^60^ In chronic lymphocytic leukemia, a correlation is also seen between Pim-1 expression and CXCR4 surface expression.^62^ As in AML, Pim-1 phosphorylates Serine339 on CXCR4.^62^ Pim inhibition in chronic lymphocytic leukemia is capable of increasing CXCR4 internalization, reducing surface re-expression and migration to bone marrow and spleen.^62^ It is therefore plausible that Pim kinases may at least partially contribute to the upregulation of CXCR4 in the hypoxic microenvironment of MM and, by this mechanism, contribute to migration and homing of cells in MM also.

### Pim kinases and resistance to therapy

Pims have been described as having a role in anti-cancer drug resistance. This may be accomplished by increasing the expression of drug efflux pumps as in breast cancer,^63^ by altering phosphorylation status of relevant receptors, for example, androgen receptor in prostate cancer.^64^

In hematologic malignancies, targeting Pim kinases in combination with standard treatment has proved useful in overcoming resistance in preclinical models, for example, combination with JAK2 inhibition in MPN^65^ and combination with cytarabine in AML.^66^ Pim-2 confers resistance in hematopoietic cells to rapamycin inhibition of mTOR.^35, 67^ Rationale for targeting the Pim and PI3K/AKT/mTOR pathways in combination is discussed below.

It is highly likely that the hypoxic bone marrow microenvironment of MM has an important role in mediating drug resistance. Pim-1 has a role in hypoxia-induced chemoresistance in a HIF1α-independent manner by altering mitochondrial transmembrane potential and the activity of caspases-3 and -9.^68^ Knockdown of Pim-1 resensitizes cells to chemotherapy.^68^ Treatment with bortezomib increases Pim half-life by prevention of Pim proteasomal degradation,^23^ and it is possible that by this mechanism, Pims have a role in resistance to proteasome inhibitors in MM.

## The Pim kinases as drug targets in MM

The unusual structure of the Pim kinase ATP-binding site and challenges with inhibiting Pim-2 have been discussed. Given the redundant functions of the Pim family kinases established in lymphoid malignancies, it is conceivable that MM cells are capable of upregulating Pim-1 or Pim-3 as a survival mechanism if Pim-2 alone is targeted, and pan-Pim inhibition is most likely to be useful in the clinic. Structure- and property-based design approaches are actively used to identify inhibitors capable of targeting all Pim family members with potency in the nanomolar range.^15, 16^

### Pim kinase inhibitors in development in MM

#### LGH447

LGH447 is a potent pan-Pim inhibitor which has entered clinical phase development in MM (See Table 1). *In vitro* work in MM cell lines revealed cytotoxicity in Pim2-dependent MM by inhibiton of mTORC1 with reduced phosphorylation of 4EBP1 and S6K; promoting apoptosis with inhibition of BAD phosphorylation and cleavage of PARP and caspases 3, 7, 8 and 9; and cell cycle arrest at G_0_/G_1_.^52, 53^ *In vitro* work has also demonstrated an inhibitory effect of LGH447 on OCs, inhibiting their formation via reduced expression of phospho-ERK1/2 and NFATc1, and inhibiting resorptive function by reducing expression of matrix metalloproteinase, carbonic anhydrase II and the F actin ring necessary for functionality.^53^ Furthermore, reduced tumor growth and bone tumor burden was seen in human xenograft MM models following treatment with LGH447.^52^ LGH447 is active in the VK*MYC transgenic mouse model,^69^ which exhibits Myc dysregulation and is highly faithful to human MM, boasting positive and negative predictive values of 67% and 86%, respectively, for clinical effective of anti-MM treatments.^70^

Results of a first-in-human Phase I study of LGH447 in relapsed/refractory MM (NCT01456689) have recently been reported.^71^ Fifty-four patients received LGH447 as monotherapy with a median of four lines of prior treatment and 68.5% had been previously treated with both proteasome inhibitors and Imids. Response was evaluated in 48 with overall response rate (partial response or greater) 10.5%, clinical benefit rate (minor response or greater) 21.1% and an impressive disease control rate (stable disease or greater) of 71.9%. Maximum tolerated dose was 500 mg daily. Dose-limiting toxicities observed were grade 3/4 thrombocytopenia (4), grade 3 fatigue (2), grade 3 hypophosphatemia (1) and vasovagal syncope (1). Most adverse events were grade 1/2 and manageable, and no deaths occurred during the study.^71^ A phase I/II clincial trial of LGH447 in combination with the PI3K inhibitor BYL719 in relapsed/refractory MM is ongoing (NCT02144038).

#### LGB321

This potent ATP-competitive pan-Pim kinase inhibitor has activity against a broad range of hematologic malignancy cell lines including Pim-2-dependent MM.^72^ LGB321 was identified during a screen for pan-Pim inhibitors and selected for further development because of a relative potency for inhibiting Pim-2 with its lower *K*_m_ for ATP.^16^ Mechanistic studies indicate its cytotoxic anti-MM effect is mediated by inhibition of mTORC1 signaling and BAD phosphorylation.^72^ LGB321 was effective in a Pim-2-dependent MM xenograft model.^1^ To date, LGB321 has not entered clinical development.

#### AZD1897

This ATP-competitive pan-PIM kinase inhibitor has been studied in *in vitro* and *in vivo* settings in combination with Akt inhibitor AZD5363 in MM and AML.^73^ In MM, AZD1897 alone reduced proliferation of MM cell lines but did not have a dramatic effect on cell death, whereas in combination with the Akt inhibitor synergystic cytotoxicity was observed, with >50% cell death. Convergence on inhibition of BAD and MCL1 are the mechanism of synergystic cytotoxicity, as well as enhanced inhibition of mTOR signaling.^73^ AZD1897 has not yet reached clinical phase development.

## Rational combination strategies for Pim inhibitors

As well as potential for combination with current standard of care in an attempt to overcome resistance/refractoriness, there is strong rationale for investigating Pim inhibitors in combination with inhibitors of the PI3K/AKT/mTOR pathway and MAPK/ERK pathway inhibitors.

### PI3K/AKT/mTOR pathway

This pathway is highly expressed in MM and shares many downstream targets with the Pim kinases which function in parallel, though independently. In MM, the salient overlapping function is mTORC1 regulation. Pim kinases phosphorylate TSC2 to activate mTORC1 and also phosphorylate 4EBP1 with activation of EIF4E resulting in cap-dependent translation.^1^ Akt signaling via PRAS40 exerts the same effect on mTORC1.^1^ Following this, not only is cell proliferation enhanced, apoptosis is also indirectly prevented by upregulation of MYC and MCL-1. Both pathways phosphorylate BAD to prevent apoptosis also. Pim kinases are capable of mediating resistance to the targeting of the PI3K/AKT/mTOR pathway.^35, 67, 74^ In lymphoid malignancies, Pims allow cells to maintain mTORC signaling in the context of mTOR inhibition.^67^ In prostate cancer, resistance to AKT inhibition is mediated by Pim-1 increasing translation of receptor tyrosine kinases in a cap-independent manner.^74^ Combinations of PI3K inhibitors and Pim inhibitors in MM *in vitro* work have shown synergy,^1^ and a recent abstract indicates synergistic cytotoxicity with the combination of AKT inhibitor AZD5363 and Pim inhibitor AZD1798 with convergence on inhibition of BAD and MCL-1 signaling outlined as key mechanisms,^75^ as was previously demonstrated in AML.^73^ This provides clear rationale for pursuing this combination into clinical studies.

### MAPK/ERK pathway

This pathway is constitutively active in MM and implicated in MM-related bone disease.^76^ Targeting this pathway proved effective in reduction of tumor burden in murine models.^77^ MAPK family members including ERK and JNK are upregulated in response to Pim signaling in prostate cancer,^12^ AML,^78^ hematopoietic cells^79^ and cardiomyocytes.^80^ The combination of Pim inhibition and MAPK inhibiton affected synergystic cell killing in T-cell leukemia.^81^ The risk of cardiotoxicity may be significant with this combination given the known risk of QTc prolongation with Pim inhibitors and the importance of MAPK signaling related to Pim expression in cardiomyocytes.

## Future directions

Much progress has been made in the treatment of MM to date, however, novel treatment approaches are urgently required to improve patient survival. It is hoped that further strides toward cure will be realized with emerging treatments such as Pim inhibition.

Knowledge of MM, and of the biology of cancer in general, dictate that in response to small molecule inhibition of kinase signaling pathways, compensatory signaling can affect resistance. The Pim kinases are likely to prove no exception. Murine models of AML have demonstrated that a high tumor burden persists in spite of improved survival with Pim inhibition with rapid relapse on drug withdrawal^12^ suggesting that disease control, rather than significant tumor killing, is the realistic effect of Pims as single agents. The greatest effect of Pim inhibition in MM is on proliferation, which is unlikely alone to result in total elimination of the malignant clone. It is therefore expected that combination approaches will be necessary for Pim inhibition to provide significant survival benefit.

Combination of Pim inhibitors with current standard therapies capable of affecting cell death may prove an effective strategy. It is known that proteasome inhibition prevents degradation of Pim kinases,^23^ lending rationale to combination of Pim inhibitors with proteasome inhibitors, a key standard component of MM treatment regimens.

Pim expression in MM correlates with advanced disease, and clinical studies of LGH447 in relapsed/refractory MM are ongoing with data reporting impressive single agent activity.^71^ Other survival signaling pathways are simultaneously active in advanced disease and combining Pim inhibition with inhibitors of the PI3K/Akt/mTOR pathway in this setting constitutes another application for Pim inhibitors in MM. Where TORC1/TORC2 inhibitors have failed to translate to extended survival in the clinic, Pim kinases, particularly as combination treatments, may have more success. The Pim kinases themselves mediate resistance to mTOR inhibition in hematologic malignancies and may partly account for the lack of efficacy encountered.^35, 67^ The converse may also be true, and it may prove necessary to inhibit the PI3K/Akt/mTOR pathway in tandem for durable results. Pim inhibition may have an added advantage over mTOR inhibition given the myriad other MM processes Pims are involved in.

In general, Pim inhibitors demonstrate good tolerability and manageable side effects in the data reported.^71^ Avoidance of overlapping toxicities is a priority in the selection of suitable Pim inhibitor drug partners, with the propensity of Pim inhibitors to induce cardiotoxicity a key consideration. As inhibiting proliferation is the key to the anti-MM activity of Pim kinases, and can be achieved at doses lower than those required for induction of apoptosis, for example, utilizing the minimum dose required to achieve this anti-proliferative effect may limit potential for cardiotoxicity. The Pim inhibitors are also capable of suppressing hematopoiesis and selection of combination partners from currently available treatments should focus on less myelosuppressive agents.

Ongoing clinical studies of LGH447 as a single agent and in combination with PI3K inhibitor BYL719 in advanced MM are anticipated to expedite the progression of Pim inhibitors with rational partners to the clinic and are eagerly awaited.

## Figures and Tables

**Table 1:** Pim inhibitors in development

*Drug*	*Class*	*Phase of development*	*Mechanism of action MM*	*Potency*	*Clinical trial identification*	*Condition*	*Status*	*Endpoint*	*Salient reports*	*Ref.*
LGH447	3-S-aminopiperidine pyridyl carboxamide	Phase I/II	-Reduces p4EBP1, pS6K -Reduces pBAD -Cleave PARP and caspases 3,7,8,9 -Cell Cycle Arrest Go/G1	Ki Pim-1 5.8pM; Pim-2 18.0pM; Pim-3 9.3pM	NCT01456689	RRMM	Recruiting	MTD RDE	MTD 500 mg od ORR 10.5% CBR 21.1% DCR 71.9%	^52, 53, 71^
					NCT02160951	RRMM	Recruiting	MTD RDE	Not reported at time of writing	
					NCT02144038	RRMM, in combination with PI3K inhibitor BYL719	Recruiting	MTD RDE	Not reported at time of writing	
					NCT02078609	AML/ high risk MDS	Recruiting	MTD	Not reported at time of wristing	
AZD1208	Thiazolidene	Phase I (terminated)	-Reduces p4EBP1, pS6K -Reduces pBAD	IC50 <5 nM for each isoform	NCT01489722	AML	Terminated	MTD Efficacy	Not for further investigation owing to safety/efficacy issue	^82^
					NCT01588548	Advanced lymphoma and solid organ malignancies	Completed	MTD		
SGI-1776	Imidazopyridine	Phase I (terminated)	-Reduces p4EBP1, pS6K -Reduces pBAD -Autophagy	IC50 Pim-1 7 nM; Pim-2 363 nM; Pim-3 69 nM	NCT00848601	Refractory Prostate cancer/ RR NHL	Terminated	MTD PK PD	Terminated owing to cardiotoxicity	^39^
					NCT01239108	RR Leukemia	Withdrawn	MTD	Terminated owing to cardiotoxicity	
LGB321	3- S-aminopiperidine pyridyl carboxamide	Preclinical	-Reduces p4EBP1, pS6K -Reduces pBAD	Ki Pim-1 1pM; Pim-2.1pM; Pim-3 0.8pM	N/A		N/A	N/A	N/A	^1, 16, 72^
AZD1897	Thiazolidene	Preclinical	-Reduces p4EBP1, pS6K	Data not available	N/A		N/A	N/A	N/A	^75^

Abbreviations: AML, acute myeloid leukemia; CBR, clinical benefit rate; DCR, disease control rate; MTD, maximum tolerated dose; IC50, inhibitory concentration 50; Ki, inhibitory constant; MDS, myelodysplastic syndrome; ORR, overall response rate; RDE, recommended dose for expansion; RRMM, relapsed/refractory multiple myeloma; RR NHL, relapsed/refractory nonHodgkin lymphoma.
